# Is *Helicobacter pylori* Infection a Risk Factor for Prostatitis? A Case-Control Study in a Referring Tertiary Care Center

**Published:** 2016

**Authors:** Alireza Abdollahi, Masoud Etemadian, Saeed Shoar, Zohreh Nozarian

**Affiliations:** 1 *Dept. of Pathology, Imam Khomeini Hospital Complex, Tehran University of Medical Sciences, Tehran, Iran*; 2 *Dept. of Urology, Hasheminejad Hospital, Iran University of Medical Sciences, Tehran, Iran*; 3 *Development Association for Clinical Study (DACS), Student Scientific Research Center (SSCR), Tehran University of Medical Sciences, Tehran, Iran*

**Keywords:** Chronic Prostatitis, *H. pylori* infection

## Abstract

**Background:**

The optimal treatment is not possible yet for chronic prostatitis due to the unknown etiology of the diseases. We aimed to investigate the association of *Helicobacter pylori* infection with chronic prostatitis.

**Methods:**

In this prospective case-control study that conducted in Imam Hospital Complex affiliated to Tehran University of Medical Sciences in Tehran, Iran from 2014 to 2015, patients with diagnosis of chronic prostatitis according to the criteria of National Institute of Health (NIH) were enrolled. Control group constituted of consecutive healthy patients. Blood samples were obtained for each patient and control and evaluated for serum levels of anti *H. pylori* IgG, A. Data analysis was carried out using SPSS, version 18. Values of *P*<0.05 were considered statistically significant.

**Results:**

Mean ± SD age of patients was 59.5 ± 3.08 yr in the case group and 56.88 ± 3.20 yr in the control group with no significant difference (*P*>0.05). Mean ± SD levels of anti *H. pylori* IgG and IgA in the control group were 9.36 ± 7.45 U/ml and 6.25 ± 7.29 U/ml, respectively compared with 20.94 ± 16.98 U/ml and 18.63 ± 15.65 U/ml in the case group, respectively both of which revealed statistically significant (*P*<0.05).

**Conclusion:**

Chronic prostatitis is associated with *H. pylori* infection. Both anti *H. pylori* IgG and IgA are increased in patients with chronic prostatitis. Therefore, treatment of HP infection could be effective in the prostatitis cure.

## Introduction

Chronic prostatitis/chronic pelvic pain syndrome (CP/ CPPS) entitled discomfort in pelvic or perineal region is one of the most troublesome complaints in urology with an estimated incidence between 2% and 14% of men worldwide (1-3). Despite improvements in understanding of CP within the last decades (4), the exact etiology is still unclear. However, infections and inflammations have received tremendous interests in the last years changing the face of therapeutic approaches to this health resource-consuming problem (5). 

The role of microorganism in causing CP/CPPS has been widely addressed (6-7). One-step further, proinflammatory cytokines including have been found in elevated levels in prostatic secretions. Moreover, interleukin-1 (IL-1), IL-6 and tumor necrosis factor- α (TNF- α) increase in their serum levels in association with immune-mediated vascular disorder (8-9). On the other hand, anti *H. pylori* antibodies have been elevated in other tissue disorders including respiratory and cardiac diseases (8-10). Immune responses evoked by *H. pylori* infection may be associated with inflammatory reactions in prostatitis (11-12). However, not much later, the topic was forgotten and to the best of our knowledge, no more studies have been designed after the first initiation in 2010. On the other hand, some studies have referred to connections between *H. pylori* infection and other diseases like coronary diseases, cancer and gastric ulcer (8-10).

Our study aimed to investigate the relationship between anti *H. pylori* IgG, IgA, and presence of chronic prostatitis in a tertiary care center in Iran. The significance of this study is in the fact that if the connection between the *H. pylori* infection and prostatitis is proven, the latter could be treated and the patients would take less medication like some antibiotics. 

## Materials and Methods

**Patients Population**

A prospective case-control study was conducted in Imam Hospital Complex affiliated to Tehran University of Medical Sciences in Tehran, Iran from 2014 to 2015. Patients with diagnosis of chronic prostatitis according to the criteria of National Institute of National Institute of Health (NIH) (2, 13) were enrolled. Inclusion criteria were as age between 18 and 65 yr old, presence of chronic prostatitis symptoms for a minimum of 6 months (pain in lower abdomen, groins, genitalia, or sacral regions, erectile dysfunction or painful ejaculation and sings of inflammation on prostatic secretions i.e. presence of white blood cells or polymorph nuclear cells), not remarkable urological assessments, negative results for microbiological studies, no signs of inflammatory reactions including erythrocyte sedimentation rate (ESR), and normal liver and renal function test. Control group constitutes of consecutive healthy patients attending outpatient clinic of in Imam Hospital for routine laboratory check-ups. 

In the control group, no urologic and symptomatic digestive disease was reported. They had been reported as healthy after their checkup at lab. The case and control groups were picked as to match in age and social conditions.

Patients with any history of previous urological surgeries, those with known status of urinary tract infections or other disorders, laboratory findings of renal (Cr>1.8mg/dl) or liver (increased liver enzymes including AST, ALT, ALKP) dysfunctions, and patients undergone anti *H. pylori* eradicating medications or anti-acid drugs within the past 12 months were excluded.

**Patients Evaluation**

 All patients underwent complete physical examination as well as abdominopelvic sonography with special focus on urinary tract and genital system including prostate and surrounding structures. Additional tests were requested in case of any clinical impressions.

**Sampling**

 Four ml blood samples were obtained from peripheral intravenous (IV) lines for each patient. Specimens were transferred to the central laboratory to be evaluated in terms of complete blood counts (CBC), prostatic specific antigen (PSA), serum levels of anti *H. pylori* IgG and IgA in accordance with the manufacturer’s guidelines (German Immunolab kits).

**Statistical Analysis**

 Data analysis was carried out using SPSS ver. 18 (Chicago, IL, USA). Chi Square test for qualitative variables and student *t* test for Quantitative variables were employed and the values of *P*<0.05 were considered statistically significant. 

## Results

 A total of 126 patients entered this prospective study of which 42 patients (33.3%) had the diagnosis of CP/CPPS (case group) and 84 patients (66.7%) were healthy subjects (control group). Mean ± SD age of patients was 59.5 ± 3.08 yr in the case group compared with 56.88 ± 3.20 yr in the control group with no significant difference (*P*>0.05) (Table1). Most patients aged between 55 and 60 yr old. All patient of case group be positive for *H. pylori* infection.

**Table 1:** Comparing anti-HP anti-body titer in prostatitis patients and control group

	**Case Group (n=42)**	**Control Group (n=84)**	***P*** ** value**
**Age (yr) **	59.5±3.08	56.88±3.20	NS
**Anti ** ***H. pylori*** ** IgG (U/ml) **	20.94±16.98	9.36±7.45	*P*<0.0005
**Anti ** ***H. pylori*** ** IgA (U/ml)**	18.63±15.65	6.25±7.29	*P*<0.001

 Mean ± SD serum levels of anti *H. pylori* IgG and IgA were 9.36 ± 7.45 U/ml and 6.25 ± 7.29 U/ml, respectively in the control group compared with 20.94 ± 16.98 U/ml and 18.63 ± 15.65 U/ml, respectively in the case group which both revealed statistically significant increase in patients with CP (*P*<0.05).

The Hb did not differ meaningfully in the case and control groups (*P*>0.05). PSA in the case group was a bit higher than in the control group, but statistically, the difference was not meaningful (*P*>0.05).

## Discussion

 CP is a prevalent disease with a remarkable physical, mental, and socioeconomic burden (4). However, because its underlying causes is still unknown, the optimal treatment has never come possible. Recently, multimodal approaches to CP have been considered (5). Increasing evidences are relating *H. pylori* infection with urological diseases (14). Most evidently, *H. pylori* has been found to induce cystitis, a condition which may further lead to bladder lymphoma. If a relation is found between *H. pylori* and CP, then eradication of this organism may proceed to prevent such urological disease just like what anti *H. pylori* did in peptic ulcer diseases (14). Although several studies have investigated the role of infectious organisms in CP and other lower urinary tract diseases (1, 3-7, 14), there are little evidences existing about the causative role of *H. pylori* in CP. 

 Karatas et al. compared the seroprevalence of *H. pylori* infection among 64 cases of CP/CPPS and 55 asymptomatic controls (11). They found a higher seropositivity against *H. pylori* among CP patients (76%) compared with the controls (62%). Similarly, in our study, there were higher serum levels of anti *H. pylori* IgA and IgG in patients with diagnosed CP than in control patients. To date, there is no other study, which could be compared with our findings in terms of seroprevalence of *H. pylori* in CP/CPPS. However, detection of *H. pylori* antigens may relate such an infection more clearly to urological disease including CP/CPPS (14). 

 CP is evoked by a primary stimulus within the prostate gland (15). Although microorganisms such as *Escherichia coli, Mycoplasma genitalium, *and* Chlamidya trachomatis* have been accused in this regard (16), it has not been well documented whether the stimulus is of definite infectious etiology (5). If the role of infectious disease in development of such a disease is accepted, *H. pylori* can be also listed as a key etiology. *H. pylori* has been resulted in elevated proinflmmatory cytokines in human body (14). On the other hand, IL-6, IL-8, IL-10, IFN-γ, and TNF-α are increased in secretions of prostate glands in patients with CP/CPPS (6). Both anti *H. pylori* IgA and IgG elevated in our CP patients compared to control group. However, IgM may not be of important cause for CP due to its chronic process and maybe question the role of *H. pylori* infection in CP to some degrees.

*H. pylori* organism may cause prostatitis from different ways. For instance, the organism may directly enter the prostate and cause disease or penetrate stomach and excrete cytokines and interleukins, which would be circulated in the blood into the prostate and cause inflammation or the *H. pylori* infection does reduce the body’s immunity and the prostate would be affected. The small size of the sample, non-detected of antigen *H. pylori* and its culture were among the restrictions in this study.

To our knowledge, this is the second study to show the relationship between *H. pylori* and CP following a pilot study by Karatas and colleagues (11). Although our study presented more quantitative results compared with previous one by comparing mean serum levels of anti *H. pylori* IgA and IgG between the two groups, there is still a paucity of evidence regarding the definite role of *H. pylori* in CP. Quantitative measurements of microorganism antigens by PCR may serve more efficiently to confirm our findings. Furthermore, larger sample size and multicenter design would be additive values of further studies. 

The significance of this study is in the fact that if the connection between the *H. pylori* infection and prostatitis is proven, the latter could be treated and the patients would take less medication like some antibiotics. 

## Conclusion

 Chronic prostatitis may be associated with *H. pylori* infection. Both anti *H. pylori* IgG and IgA are increased in patients with chronic prostatitis.
